# Assessing Older Adults’ Frequency of Going Outside and Physical Performance: NHATS 2017

**DOI:** 10.1093/geroni/igaa057.2876

**Published:** 2020-12-16

**Authors:** Justine Sefcik, Janeway Granche, Martha Coates, Zachary Hathaway, Rose Ann DiMaria-Ghalili

**Affiliations:** Drexel University, Philadelphia, Pennsylvania, United States

## Abstract

Little is known about community-dwelling older adults’ outdoor activity and the relationship between physical function and frequency of going outside. Using the 2017 NHATS (N = 4,465), we looked at self-reported outdoor frequency (Likert scale: every day to once a week or less) and the Short Physical Performance Battery (SPPB; participants completed five different physical activities to measure physical performance; total scores ranged from 0, not attempted, to 12, the best). A logistic model comparing community-dwelling older adults going out most days (18.3%), some days (10.3%), or rarely/never (3.4%) to those going out every day found ORs of 0.85, 0.70, and 0.58 respectively (all p<0.0001) for a one-unit increase in SPPB score. Interdisciplinary teams can use findings to assess disabled community-dwelling older adults’ frequency of going outdoors. Implications for interventions to assist with increasing times leaving the home (e.g. mobility devices, caregiver assistance) will be discussed.

